# Unveiling the Nottingham Inversion Instability during the thermo-field emission from refractory metal micro-protrusions

**DOI:** 10.1038/s41598-021-94443-7

**Published:** 2021-07-26

**Authors:** Darius Mofakhami, Benjamin Seznec, Tiberiu Minea, Romaric Landfried, Philippe Testé, Philippe Dessante

**Affiliations:** 1 Laboratoire de Génie Electrique et Electronique de Paris, Université Paris-Saclay, CentraleSupélec, CNRS, 91192 Gif-sur-Yvette, France; 2 Laboratoire de Génie Electrique et Electronique de Paris, Sorbonne Université, CNRS, 75252 Paris, France; 3 Laboratoire de Physique des Gaz et des Plasmas, Universite Paris-Saclay, CNRS, 91405 Orsay, France

**Keywords:** Electrical and electronic engineering, Electronic properties and materials, Nonlinear phenomena

## Abstract

The electron emission by micro-protrusions has been studied for over a century, but the complete explanation of the unstable behaviors and their origin remains an open issue. These systems often evolve towards vacuum breakdown, which makes experimental studies of instabilities very difficult. Modeling studies are therefore necessary. In our model, refractory metals have shown the most striking results for discontinuities or jumps recorded on the electron emitted current under high applied voltages. Herein, we provide evidence on the mechanisms responsible for the initiation of a thermal instability during the field emission from refractory metal micro-protrusions. A jump in the emission current at steady state is found beyond a threshold electric field, and it is correlated to a similar jump in temperature. These jumps are related to a transient runaway of the resistive heating that occurs after the Nottingham flux inversion. That causes the hottest region to move beneath the apex, and generates an emerging heat reflux towards the emitting surface. Two additional conditions are required to initiate the runaway. The emitter geometry must ensure a large emission area and the thermal conductivity must be high enough at high temperatures so that the heat reflux can significantly compete with the heat diffusion towards the thermostat. The whole phenomenon, that we propose to call the *Nottingham Inversion Instability*, can explain unexpected thermal failures and breakdowns observed with field emitters.

## Introduction

Modern and future ultra-high-voltage vacuum equipment requires ever better electrical insulation. This is especially true for particle accelerators^1,2^ and neutral beam injectors for tokamaks^3^, as well as other high-voltage direct current devices. Unfortunately, under exceptionally high applied electric fields ($10^7$–$10^9$ V/m), several well-identified physical phenomena can cause a series of interdependent events often evolving towards a vacuum breakdown. Most of these breakdown events are related to the presence on the cathode of micro/nano-protrusions, whose shape and density depend on the surface roughness^4^. Besides, such protrusions may also emerge and evolve *via* electromigration^5^ and field-induced surface atom diffusion^6^. Locally enhancing at their apex the high applied electric field, these protrusions act as field electron emitters. Above a certain field magnitude, the current density inside these emitters becomes high enough to generate self-heating *via* a combination of both resistive heating in the emitter volume (Joule heating) and the Nottingham effect at the emission surface^7,8^.

Let us recall the Nottingham effect comes from the energy balance between the mean energy of the emitted electrons $\langle \epsilon _\text {out} \rangle$ and that of the replacing electrons $\langle \epsilon _\text {in} \rangle$, the so-called Nottingham energy $W_\text {N}=\langle \epsilon _\text {in} \rangle -\langle \epsilon _\text {out} \rangle$. It yields a heat flux at the metal/vacuum interface whose magnitude depends on the emitter current density *J*, according to the formula $\Phi _\text {N}=- W_\text {N} \times J / e$ where *e* is the elementary charge. Therefore, the sign of this heat flux can reverse. At a given field magnitude, the heat flux is positive (heating) below a certain temperature and reverses above, becoming negative (cooling). The inversion temperature is called the *Nottingham temperature* and is analytically found proportional to the local electric field magnitude and inversely proportional to the square root of the emitter work function^7^: $T_\text {N}\propto F/\varphi ^{1/2}$.

Because higher temperatures facilitate the emission of electrons, it is well established that the self-heating of an emitting protrusion may lead to a vacuum breakdown, following different possible scenarios. If the temperature at the emitter apex reaches the melting point, vapor will be released. Its ionization by the energetic emitted electrons in front of the protrusion can then ignite an arc, possibly yielding a breakdown^9,10^. Another, less frequent, possibility could be the explosion of the protrusion, namely with RF fields^11,12^, due to a much faster increase of the temperature and/or the maximum temperature reached below the apex, in the emitter volume^13^. On the other hand, a high thermo-field current from the cathode can also locally heat the anode and cause the detachment of adsorbed microparticles, which then collide the cathode (the micro-protrusions), following the Cranberg scenario^14–16^.

Similar thermal instabilities during field emission are also an issue for modern electron sources’ design. Since the last decades, efficient vacuum electron sources based on optimally spaced arrays of sharp field emitters purposely grown at a cathode surface offer higher integrability, commutability and durability than their thermionic counterparts^17–19^. However, the emitter self-heating during thermo-field emission limits the operation voltage, capping the emitted current performances^20–23^. Hence, the question of thermal stability in the self-heating process of field-emitting protrusion is an issue for both the domain of field electron sources and high-voltage vacuum insulation.

Unfortunately, it is technologically very difficult—even impossible—to perform reproducible experiments close to the breakdown while locally track the heat-related quantities at the individual emitter scale. Besides, the damages caused onto the system in case of a breakdown generally have critical consequences. Therefore, analytical works have been conducted in parallel with the experiments to get general insights into the self-heating process^24–27^. Nevertheless, considering the problem complexity—related to the solving on a real geometry of the coupled equations of heat and current—many simplifying assumptions are necessary. Most of the published solutions reduce the system to only one dimension (1D), often stationary, and not or only partially taking into account the Nottingham effect—Notice that the Nottingham effect varies with the surface temperature, making the equation system self-consistent, since the boundary condition (the Nottingham heat flux) is function of the solution (the surface temperature). Therefore, we should rather focus our overview on multi-physics model of the self-heating process.

Relying on the ever-increasing computing power, several numerical models have followed one another since the ’80s. They brought insights into the heating evolution in time^28–30^ along with more accurate considerations of the underlying quantum dynamics^31–33^. Concerning the contribution of the Nottingham effect to the emitter self-heating, modeling works have shown that it was predominant over the resistive heating at low current density, while the situation reverses at higher density^34,35^. Once the apex temperature exceeds the Nottingham inversion temperature, the cooling Nottingham effect even causes the maximum temperature to sink beneath the surface, as highlighted by Fursey^13^ and Rossetti et al.^36^. However, these studies overall lacked a detailed description in time of the various heating terms and an exhaustive exploration of the applied voltage (or electric field).

To go further, our work investigates the self-heating evolution during the electron emission from single protrusions. For each protrusion, the whole thermo-field regime is explored, up until the melting point is reached. The focus goes on an unstable thermal behavior of refractory metal protrusions related to the Nottingham heat flux inversion. Once a threshold electric field is exceeded, a runaway of the self-heating process occurs, drastically facilitating the emitter thermal failure or even directly causing it. The whole phenomenon, not documented so far, is proposed to be called the *Nottingham Inversion Instability*. It is shown to be caused by threshold feedback mechanisms that depends on the emitter geometry and specific material properties. Thus, besides its contribution to the domain of electron sources and vacuum breakdowns, this work sheds some light on the possible nonlinear evolution of self-consistently coupled equations, which present a fundamental interest in the field of complex system dynamics.

## Emission model

**Figure 1:**
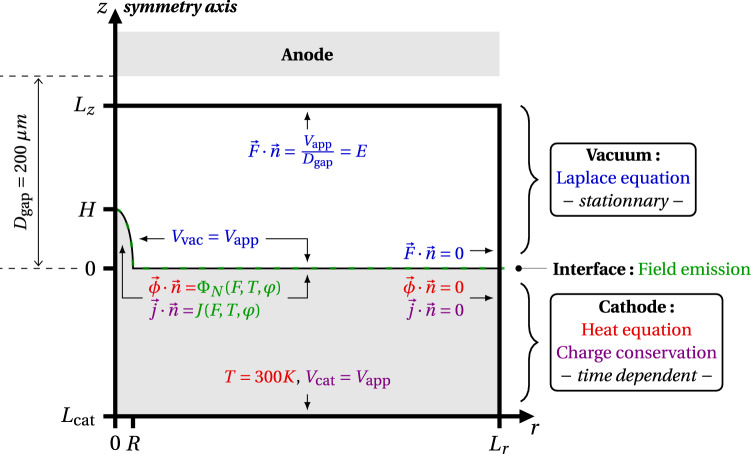
Scheme of the simulation domain in a 2D sectional view along the radial coordinate *r* (not at scale). The domain is bound between $[L_\text {cat}, L_z]$ along the symmetry *z*-axis, and between $[0,L_r]$ along the *r*-axis. The boundary conditions are shown in blue for the Laplace equation (solved in the vacuum gap) and respectively in red and purple for the heat and current equations (solved in the cathode). The green dashed line highlight the cathode-vacuum interface. The field emission related quantites at the interface are highlighted in green.

Our model uses a finite element approach to reproduce any 3D geometry and a time-dependent solver based on the Backward Differentiation Formula method. In this paper, the analysis is focused on the thermal evolution of single axially symmetric protrusions, located on a plane cathode and facing a plane anode (Fig. 1). The applied voltage is denoted $V_\text {app}$ and a time ramp is set so that $V_\text {app}(t)=V_\text {app}\left[ 1-\exp (-t/\tau )\right]$ with the time constant $\tau =1~ns$ to correctly simulate the initiation of field emission. The corresponding applied electric field is deduced from the distance $D_\text {gap}=200~\upmu$m between the cathode and the anode: $E=V_\text {app}/D_\text {gap}$. The local electric field *F* at the emitter surface is obtained by solving the Laplace equation in the vacuum gap (space-charge is not taken into account) :

1 $$\begin{aligned} \Delta V_\text {vac}=0 \, , \quad \vec {F}=-\vec {\nabla } V_\text {vac} \end{aligned}$$

where $V_\text {vac}$ is the voltage inside the vacuum chamber. The field enhancement factor at the emitter apex is denoted $\beta$, so that the local field there writes $F_\text {a}=\beta E$.

Together with the material work function $\varphi$ and the temperature *T* (initially at room temperature), the field magnitude is used to numerically compute the emitted current density $J(F,T,\varphi )$ by integrating over all normal energy the product between the supply function and the transmission probability. The supply function is obtained within the framework of the Sommerfeld theory while the transmission probability is computed following the Kemble formalism^37^, assuming a 1D potential barrier corrected by the image charge: the so-called Schottky–Nordheim Barrier. Detailed formulas can be found in our previous work^38^, appendix A therein. Note that the 1D approximation does not hold for curvature radii below 20 nm^30^. However, our study limits to curvature radii down to 39 nm (taking the Gaussian curvature $H/f^2$ for the hemiellipsoid with $H=10~\upmu$m and $f=16$). The Nottingham heat flux $\Phi _\text {N}(F,T)=- W_\text {N} \times J / e$ is then deduced from the emission current density *J* and the Nottingham energy $W_\text {N}$, assuming a mean energy equal to the Fermi level for the replacement electrons.

Both the Nottingham heat flux and the emission current density are used as von Neumann boundary conditions at the cathode upper surface (i.e. the vacuum–cathode interface, see the green dashed line in Fig. 1) to solve the time evolution of the coupled equations of heat:

2 $$\begin{aligned} \rho (T)c(T)\frac{\partial T}{\partial t}-\vec {\nabla } \cdot \vec {\phi } = \frac{j^2}{\sigma (T)} \, , \quad \vec {\phi } =-\kappa (T)\vec {\nabla }T \end{aligned}$$

and current:

3 $$\begin{aligned} \vec {\nabla }\cdot \vec {j}=0 \, , \quad \vec {j}=\left( \sigma (T) + \varepsilon _0\frac{\partial }{\partial t} \right) \vec {\nabla }V_\text {cat} \end{aligned}$$

where $\rho$, *c*, $\kappa$ and $\sigma$ respectively denote the volumetric mass density, the specific heat capacity, the thermal conductivity and the electrical conductivity of the cathode material, all being temperature-dependent (see “Material properties”). Note that the thermal losses due to surface radiation are negligible in all analyzed cases. At the cathode lower surface, Dirichlet boundary conditions are set assuming constant temperature and potential. Far away on the simulation box lateral boundary, symmetry conditions are enforced. At each time step, the field and temperature at the emitter surface are updated and the self-heating process is simulated until a steady state is reached or no convergence is found. All boundary conditions are shown in Fig. 1.

## Results

### Discontinuous transition between two steady states

**Figure 2:**
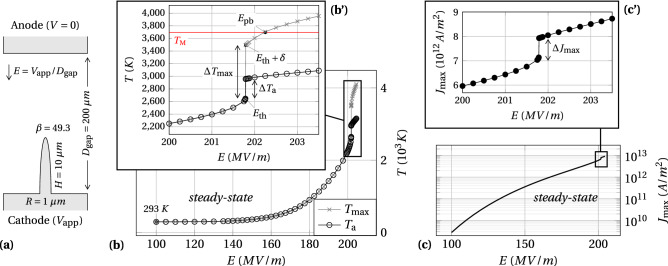
Highlight of a temperature jump versus the applied electric field in the steady state thermo-field emission of a tungsten hemiellipsoid. (**a**) Sketch of the emitter. $\beta$ is the apex field enhancement factor, so that the locally enhanced electric field at the apex is $F=\beta E$, where *E* is the applied electric field. The dimensions are not at scale. (**b**) Variation of the emitter maximum temperature $T_\text {max}$ and its apex temperature $T_\text {a}$ *versus* the applied electric field. (**c**) Variation of the maximum emitted current density $J_\text {max}$ *versus* the applied electric field. Each data point is the result *at steady state* of a transient simulation where the field is raised from zero to the indicated x-axis value over a few nanoseconds. (**b’**,**c’**) are zooms of (**b**,**c**) framed areas, respectively.

Although our model is able to deal with 3D arrangements of emitters^38^, for the sake of clarity and reduced computation time, the results presented hereafter were obtained with a single axisymmetric protrusion. Note that the highlighted phenomena would occur in a similar way with more complex 3D geometries, as long as the conditions detailed later on in the discussion are fulfilled. Let us therefore start with the case of a tungsten hemiellipsoid emitter, with the work function taken to $\phi =4.5~$ eV, height $H=10~\upmu$m and base radius $R=1~\upmu$m, which gives an aspect ratio $f=H/R=10$ and an apex field enhancement factor $\beta =49.3$ (Fig. 2a).

First, Fig. 2b shows the increase of both the temperature at the emitter apex $T_\text {a}$ and the maximum temperature inside the emitter $T_\text {max}$, at steady state, versus the applied electric field *E* . Being interested in the self-heating process, we explore fields covering all the possible range for the maximum temperature, from room temperature ($\sim 300$ K) up to the melting point of tungsten $T_\textsc {m}=3695$ K. The corresponding current densities are shown in Fig. 2c with $J_\text {max}$ being the maximum emission current density released from the emitter surface, essentially located at the apex. Surprisingly, the curves exhibit a sudden jump in both temperatures and in current density, denoted $\Delta T_\text {a}$, $\Delta T_\text {max}$ and $\Delta J_\text {max}$ respectively. These jumps occur at a certain value of the applied electric field, named hereafter the *threshold applied electric field* $E_\text {th}$. In addition, below $E_\text {th}$, the maximum temperature equals the apex one, meaning the apex is the hottest point of the emitter. However, above $E_\text {th}$ the maximum temperature significantly exceeds the apex temperature, which may sound counterintuitive at first glance since the Joule heating is still maximal at the apex. From the numerical point of view, all these solutions did converge well, but it was not possible to reach a steady state with a maximum temperature in the jump region. That is why we assimilate this jump to an instability caused by the coupled evolution of several terms in the equation system. Refining the field sampling around the threshold down to a step $\delta =$5 kV/m gives a value of $E_\text {th}=201.785$ MV/m. The related jumps in temperature are then $\Delta T_\text {max}=843$ K and $\Delta T_\text {a}=311$ K, meaning the maximum temperature separates from the apex by about 500 K. After this jump, it is important to note that the maximum temperature still has not exceeded the melting temperature. Therefore, all the simulations are physically valid up until they exceed the horizontal red line at $T_\textsc {M}=3695$ K. This occurs for a *pre-breakdown* electric field, $E_\text {pb}=202.25$ MV/m. Beyond $E_\text {pb}$, additional physics come into play that will eventually lead to a breakdown (gray crosses beyond $E_\text {pb}$, Fig. 2b-inset).

**Figure 3:**
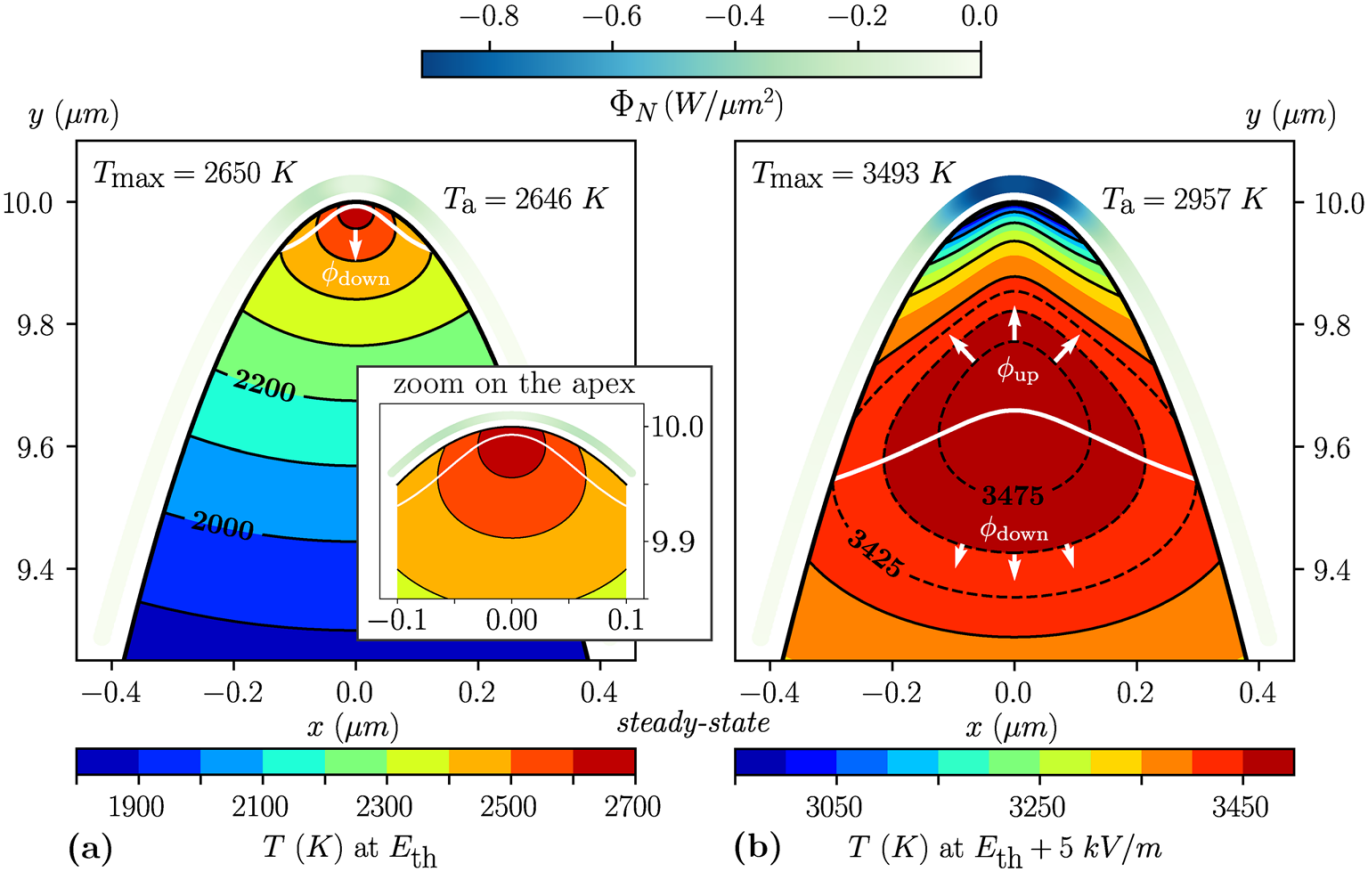
Color maps in the axisymmetric plan of both the Nottingham heat flux at the emission surface and the protrusion temperature, with the isothermal distribution. (**a**,**b**) Steady state at the threshold applied electric field $E_\text {th}=201.785$ MV/m and 5 kV/m above, respectively (see Fig. 2b’). The surface plots of the Nottingham heat flux are slightly up-shifted over the emitters for more clarity and use a common color scale for both panels (top color scale). The temperature color scales are on the contrary adapted to each panel (bottom color scales). The temperature step is 100 K between two successive solid lines and 25 K between two successive dashed lines. $T_\text {a}$ is the apex temperature. The thick white line delimits the heat flux reversal in the *z*-direction. The white arrows highlight the heat flux towards the thermostat ($\phi _\text {down}$) and the heat reflux towards the emission area ($\phi _\text {up}$).

Second, Fig. 3a,b show both the temperature and the Nottingham heat flux distributions at $E_\text {th}$ and 5 kV/m above, respectively. It is striking to see how a tiny increase of $\delta =5$ kV/m (less than $+0.0025\%$, which corresponds to a change of $+0.02\%$ in the emitted current density at 300 K) is enough to totally change these distributions. At $E_\text {th}$, the maximum temperature is 2650 K, almost identical to the apex temperature (within 4 K), and is found within a few nanometers below the apex (Fig. 2a). At $E_\text {th}+\delta$, on the other hand, the maximum temperature has reached 3493 K, exceeding the apex temperature by 536 *K*, and has sunken in the emitter volume by about 350 nm (Fig. 2b). This temperature distribution with the maximum temperature well below the apex is very similar to the distributions observed by Fursey et al. in their modeling works^13,39^ (details of their model can be found in Fursey's book^40^, subsection 3.3.3 therein). It is related to the Nottingham effect, which becomes cooling once the emitted electrons carry more energy on average than the replacement electrons. This occurs when the emission surface temperature exceeds the *Nottingham inversion temperature*, $T_\text {N}(F,\varphi )$, which depends on the material work function $\varphi$ and the locally enhanced electric field *F*. In our model, $T_\text {N}=2621$ K at the apex, where the threshold applied electric field yields $F_\text {a}=\beta E_\text {th}=9.95$ GV/m. Once the Nottingham temperature is exceeded at the emitter apex, the latter begins to dissipate heat. The maximum temperature consequently moves into the protrusion bulk. Using Fursey’s words, the detachment of the maximum temperature from the apex causes the formation of a “high-temperature domain”, where the temperature is quite homogeneously distributed, as shown in Fig. 3b (red region). The heat is then no longer entirely dissipated towards the thermostat (that is, the cathode bulk—see the boundary conditions shown in Fig. 1). A portion of the emitter volume now dissipates its heat towards the emission surface. The corresponding *heat reflux* (so-called *reflux* as it goes in the opposite direction to the thermostat) is denoted $\phi _\text {up}$, in contrast to the usual flux conducting the heat towards the thermostat, denoted $\phi _\text {down}$ (see the white arrows in Fig. 3a,b). Additionally, a thick white line marks the heat flux reversal in the *z*-direction, dividing the emitter volume into two thermally independent parts. Comparing the surface plots of Fig. 3, one can see that the magnitude of the Nottingham heat flux (top color scale) is about ten times more dissipative above the threshold field: $\Phi _\text {N}(F_\text {a},T_\text {a})=-0.09~W/\upmu$m$^2$ at $E_\text {th}$ (Fig. 3a) against $\Phi _\text {N}(F_\text {a},T_\text {a})=-0.87~W/ \upmu$m$^2$ at $E_\text {th}+\delta$ (Fig. 3b). This difference explains why the second case exhibits a significant displacement of the maximum temperature into the emitter bulk with a wide heat reflux volume. However, it does not explain why a tiny change in the electric field ($+0.0025\%$ from $E_\text {th}$ to $E_\text {th}+\delta$) yields such a thermal jump between the two steady states, with a difference in the maximum temperature above $30\%$.

### Transient runaway during the self-heating process

**Figure 4:**
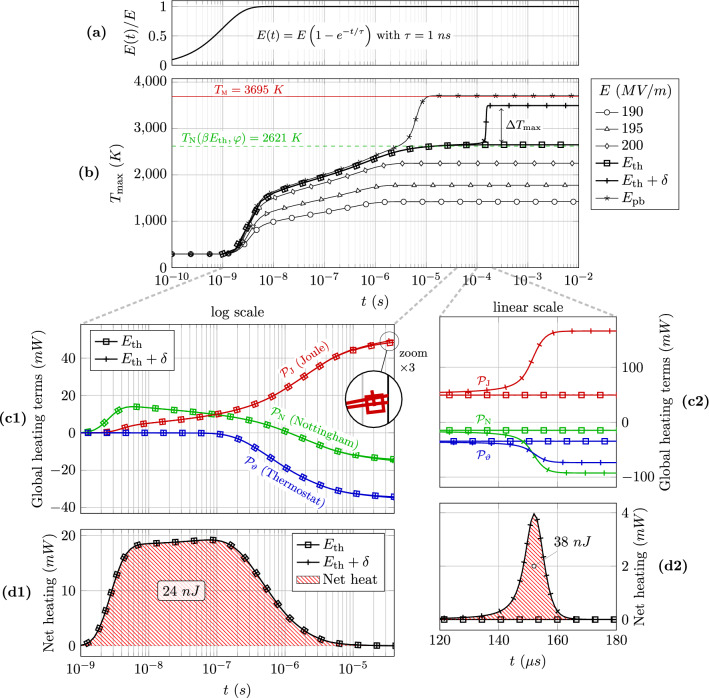
Evolution of the self-heating process. (**a**) Time ramp of the applied electric field for each simulation, normalized to 1. The time constant $\tau$ is set to one nanosecond. (**b**) Evolution of the maximum temperature during the self-heating process for different applied electric fields. The evolutions at $E_\text {th}=201.785$ MV/m and $E_\text {th}+ \delta = 201.790$ MV/m are respectively marked by square and plus signs. $E_\text {pb}=202.25$ MV/m is the pre-breakdown field. The dashed green line indicates the Nottingham inversion temperature for the specific field value $F=\beta E_\text {th}$ at the protrusion apex. The red line recalls the melting temperature of tungsten. (**c**) Detailed evolution at $E_\text {th}$ and $E_\text {th}+\delta$ of each global heating terms (**c1**) before the jump (log time scale) and (**c2**) during the jump (linear time scale). (**d**) Evolution of the net heating at $E_\text {th}$ and $E_\text {th}+\delta$ (**d1**) before the jump (log time scale) and (**d2**) after the jump (linear time scale). The net heating is the sum of the three global heating terms and its integral in time yields the net heat produced (see the red-hatched area and its label).

To better grasp the thermal gap between the two steady states around $E_\text {th}$, it is enlightening to follow on Fig. 4 the thermal balance evolution during the self-heating process. Let us recall that each transient simulation spans from $10^{-11}$ to $10^{-2}$ s with logarithmic time steps, and sets a time ramp on the electric field as shown in graph a, to simulate the electrodes response to the DC power supply.

Graph b shows the evolution of the maximum temperature for different applied electric fields. In particular, the evolutions for $E_\text {th}$ and $E_\text {th}+\delta$ are highlighted in thick black lines, respectively marked with squares and plus signs. Both curves initially follow a very similar path, up to $\sim 10^{-4}$ s when a sudden increase occurs for $E_\text {th}+ \delta$, leading to an eventually much higher maximum temperature at steady state. This deviation can be analyzed *via* the evolution of the global heating terms of the heat equation $\mathcal {P}_\text {J}$, $\mathcal {P}_\text {N}$ and $\mathcal {P}_\vartheta$ displayed in graphs c1 and c2. They respectively correspond to the integrated values of the Joule heating $j^2/\sigma$ inside the emitter volume *V*, of the Nottingham heat flux $\Phi _\text {N}$ over the emission surface $\Sigma$, and of the dissipative heat flux $\phi _\text {down}$ towards the thermostat $\vartheta$ through the emitter base:

4 $$\begin{aligned} \mathcal {P}_\text {J} = \iiint \limits _{V} \frac{j^2}{\sigma }\,\mathrm {d}V , \quad \mathcal {P}_\text {N} =\iint \limits _\Sigma \Phi _\text {N} \,\mathrm {d}S \quad \text {and} \quad \mathcal {P}_\vartheta =- \iint \limits _\text {base} \phi _\text {down} \, \mathrm {d}S \end{aligned}$$

The sum of these three terms gives the net heating, shown in graphs d1 and d2 along with its integral over time corresponding to the net heat produced (indicated as labels in the red-hatched area).

Looking first at graph c1, both electric fields exhibit a very similar evolution of the heating below $10^{-4}$ s, leading to the same production of net heat: 24 nJ (graph d1). The only noticeable difference is a very slightly higher Joule heating around $40~\upmu$s (see zoom on graph c1). This very small difference, however, drifts towards a quick runaway of the Joule heating after $10^{-4}$ s for $E_\text {th}+\delta$ as can be seen on graph c2. This runaway is then clearly damped by the usual negative feedback loop: a cooler Nottingham effect and a higher heat flux towards the thermostat due to higher temperature gradients. The resulting net heating shown in graph d2 undoubtedly highlights the runaway initiation and its consequent damping.

This is the reason why, for the considered case of a tungsten emissive protrusion, the transition from one steady state (at $E_\text {th}$) to another ($E_\text {th}+\delta$) is not continuous and is correlated with a significant gap in the accumulated thermal energy. Indeed, looking at graph d2, the curve at $E_\text {th}+ \delta$ (plus signs) highlights the production of an additional 38 nJ in $\sim 20~\upmu$s (between $t=140$ and $160~ \upmu$s) to the initial 24 nJ, while the curve at $E_\text {th}$ (square signs) already reached a steady state. Hence, a field variation of only $+0.0025\%$ causes a local overheat of $+158\%$.

Finally, for higher field variations $\delta E>\delta$, the runaway occurs sooner, develops over shorter time scales, and yields higher additional heat output. For example, at $E_\text {pb}=202.25$ MV/m (shown in graph b), the runaway triggers at $t=1~\upmu$s and brings an additional net heat of $\sim 50$ nJ over a dozen of microseconds (see Supplementary Fig. 1 online for the heating evolution). The maximum temperature then reaches the melting point, possibly initiating a vacuum breakdown. Besides, whether the field is initially set at $E_\text {th}+ \delta E$, or is ramped up by $\delta E$ after a plateau of the maximum temperature has first been reached at $E_\text {th}$, a similar runaway will develop over the same time scale and lead to the same final steady state (see Supplementary Fig. 2 online). Overall, such an abrupt deviation makes the emitter thermally unstable around the threshold field $E_\text {th}$ and facilitates the occurrence of explosive thermal failures involving material projections.

### Influence of the emitter shape and material

So far, the Nottingham effect has been shown to play a major role in the jump initiation. To better understand the underlying mechanisms causing this thermal instability, it is nonetheless necessary to study how it is influenced by the emitter parameters. After an exhaustive numerical study, we found that the most decisive parameters were the material thermal conductivity $\kappa$ and the emitter geometry. On the contrary, it was observed that the work function did not change the jump occurrence (see Supplementary Fig. 3 online). Instead, lower (respectively higher) work functions enable field emission at much lower (higher) electric field. The actual Nottingham temperature at the apex consequently decreases (increases) which essentially shifts the jump at lower (higher) temperatures, with no clear influence on its magnitude. In what follows, the work function has therefore been kept unchanged, at 4.5 eV, to facilitate comparison and reveal the importance of the other material properties.

**Figure 5:**
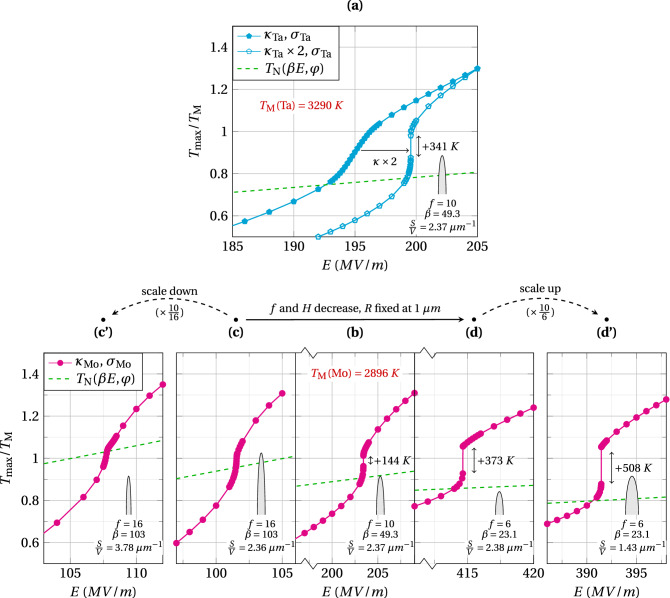
Variation of the maximum temperature with the applied electric field for selected cases. Temperatures are normalized by the material melting temperature $T_\textsc {M}$. (**a**) Case of the original emitter geometry ($H=10~\upmu$m, $R=1~\upmu$m and $f=10$) with the tantalum thermal and electrical conductivities $\kappa _\text {Ta}$ and $\sigma _\text {Ta}$, compared to the same emitter with the thermal conductivity artificially boosted, $\kappa _\text {Ta}\times 2$. (**b**) Case of the original emitter geometry with the molybdenum thermal and electrical conductivities, $\kappa _\text {Mo}$ and $\sigma _\text {Mo}$. (**c**) Sharper emitter with $f=16$ obtained by increasing the height. (**c’**) Sharper emitter with $f=16$ obtained by decreasing the radius. (**d**) Rounder emitter with $f=6$ obtained by decreasing the height. (**d’**) Rounder emitter with $f=6$ obtained by increasing the radius. The amplitudes of the temperature jumps are given with the threshold electric field refined down to a step of $\delta =5$ KV/m. The variation of the Nottingham temperature $T_N$ with the local electric field at the emitter apex $F_a=\beta E$ is also shown on all graphs. All simulations have been performed with the same work function $\varphi = 4.5$ eV.

**Figure 6:**
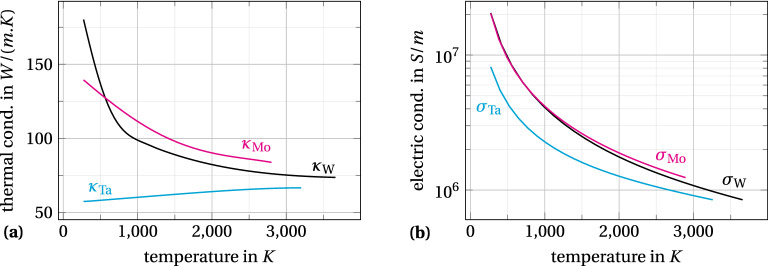
Plot of the fitting polynomials used in our model for (**a**) the thermal conductivity and (**b**) the electrical conductivity. References are given in the text.

Figure 5 compares the thermal stability of purposely selected cases, showing the increase of the maximum temperature at steady state with the applied electric field. The first case considers a tantalum emitter with the same geometry as before: $H=10~\upmu$m and $f=10$. However, the thermal and electrical conductivities of tantalum $\kappa _\text {Ta}$ and $\sigma _\text {Ta}$ are about twice lower than these of tungsten (see Material properties, Fig. 6). The transition through the Nottingham inversion temperature is then continuous as shown by the cyan curve with filled pentagons in graph a. In this case, increasing the applied electric field still yields a cooler Nottingham effect and causes the maximum temperature to sink deeper. Nevertheless, all maximum temperature locations along the emitter axis are stable and can be reached in steady state. Artificially increasing the thermal conductivity by a factor of 2 brings back a temperature jump, as depicted by the cyan curve with empty pentagons in graph a ($\kappa \times 2$). This supports a causal link between the thermal conductivity and the emitter instability at the threshold field.

Now switching to molybdenum, the last cases are focused on the influence of the emitter geometry. Compared to tantalum, the conductivites $\kappa _\text {Mo}$ and $\sigma _\text {Mo}$ of molybdenum are much closer to those of tungsten (see Material properties, Fig. 6). In this case, graph b of Fig. 5 shows that the emitter with $f=10$ does exhibit a temperature jump, which is still associated with a transient runaway of the Joule heating. The jump is, however, smaller than with tungsten: although the thermal conductivity of molybdenum is slightly higher than that of tungsten around the Nottingham temperature, it is also the case for the electrical conductivity by approximately $10\%$. Therefore, all else being equal, the molybdenum emitter generates $\sim 10\%$ less Joule heating that results in a quicker damping of the Joule runaway and explains the lower temperature jump.

Concerning the influence of the emitter geometry, the aspect ratio is expected to have the most significant impact on the emission. Indeed, for hemiellipsoid emitters, the solution of the scale-invariant Laplace equation only depends on the aspect ratio, which fully determines the electric field distribution at the emitter surface. The question, however, arises as to whether varying the aspect ratio *via* the radius or the height impacts the temperature jump the same way. The answer lies in the surface-to-volume ratio $\frac{S}{V}$, which affects the self-heating process of emitters: a lower surface to volume ratio benefits the Joule heating at the expanse of the heat diffusion, and inversely. For hemiellipsoid, this ratio writes :

5 $$\begin{aligned} S=\pi R^2 \left( 1+\frac{H}{R}\frac{\arcsin (e)}{e} \right) , \quad V=\frac{2\pi }{3}R^2H, \quad \frac{S}{V}=\frac{3}{2}\left( \frac{1}{H} + \frac{1}{R}\frac{\arcsin (e)}{e}\right) , \quad e=1-\frac{1}{f^2} \end{aligned}$$

Where *e* is the eccentricity and is close to one in the cases where aspect ratios *f* are well above one. Therefore the surface to volume ratio appears both inversely proportional to *H* and *R*. Yet, the height being well above the radius in our cases, varying the height impacts the surface to volume ratio much less than varying the radius.

Thus, at roughly constant surface to volume ratio $\frac{S}{V}\simeq 2.4~\upmu$m$^{-1}$, graph c shows that a higher aspect ratio (compared to graph b) of $f=16$ suppresses the jump: the temperature increase with the electric field is steep, yet continuous. On the other hand, graph d shows that a lower aspect ratio of $f=6$ significantly heightens the temperature jump. Although the link between the hemiellipsoid aspect ratio and the whole range of possible emitter geometry is limited, the results are still instructive: overall, a higher aspect ratio for hemiellipsoids implies a sharper electric field distribution and a smaller emission surface around the apex (see our previous work^38^, Fig. 6 and 7 therein). These two elements thus appear to act against the transient runaway of the Joule heating.

Finally, graph c’ and d’ highlights the influence of the surface to volume ratio. They have the same aspect ratio as graph c and d, respectively, but are obtained by varying the radius instead of the height. Graph c’ shows that a higher surface to volume ratio than graph c tempers the self-heating: the temperature increase with the electric field gets more gradual. On the contrary, at $f=6$, the smaller surface to volume ratio of graph d’ further amplifies the temperature jump already observed in graph d and decreases the threshold electric field. Altogether, these graphs recall that thermal failures are more likely to occur with bigger emitters, which favor self-heating: the lower surface to volume ratio benefits the Joule effect at the expense of heat dissipation. Hence, scaling down bumps and asperities by any means should always help vacuum instruments withstand higher voltages. In both cases, however, scaling up or down the emitter size does not modify the occurrence of the temperature jump. To that respect, the emitter aspect ratio appears more determinant than its scale.

Besides, it is important to note that in all the cases exhibiting a jump, the underlying Joule runaway occurs shortly after the passing of the Nottingham temperature at the emitter apex, $T_\text {N}(\beta E,\varphi )$, highlighted by the green dashed line in all graphs of Fig. 5.

## Discussion

In light of our results, the transient Joule runaway and the consequent thermal jump beyond $E_\text {th}$ appears related to a positive feedback loop that we shall now identify and discuss. The most direct evidence we can draw is that the thermal jump around $E_\text {th}$ requires the inversion of the Nottingham heat flux to cooling. In our results, the inversion occurs first at the far end of the emission surface (see the Nottingham heat flux distribution in Fig. 3a). This is because, contrarily to 1D models, a 2D axisymmetric treatment of the physics (and 3D by extension) accounts for the local electric field decrease farther from the apex, which induces a lower Nottingham temperature. This is the reason why the white line in Fig. 3a,b bends upward, indicating the presence of radial heat flux components.

At higher electric field, the Nottingham heat flux eventually reverses at the apex, which causes a displacement of the maximum temperature into the emitter volume. It results in the formation of a *high-temperature domain* whose size and shape mainly depend on the emitter geometry (i.e. on the aspect ratio in the case of hemiellipsoid emitters). This domain can be defined by an isothermal curve close to $T_\text {max}$, chosen arbitrarily, around the maximum temperature location. Taking the isothermal at $0.99~T_\text {max}$ in the case of Fig. 3b, it initially has the shape of a droplet falling from the apex, then evolves towards a spheroid that we assimilate to a “hot core” (see the animation online in the supplementary material).

With this in mind, it is apparent that the detachment of the maximum temperature is related to a significant change in the temperature gradients beneath the emitting surface. Indeed, as the Nottingham effect becomes cooling, a heat transfer emerges between the hot core and the apex that we call the *heat reflux*. This heat reflux competes with the heat diffusion towards the cathode (thermostat). It is all the more significant as the thermal conductivity is high, the Nottingham effect is cooling and the emitting surface is large. If the heat reflux is high enough, it yields a temperature increase at the emitting surface that facilitates further electron emission. Higher current density then simultaneously induces a higher Joule heating and a more dissipative Nottingham effect, benefiting the heat reflux. Hence, a positive feedback loop is initiated. Besides, as the electrical conductivity is usually lower at higher temperature, the heat surplus accentuates further the temperature gradients. At the same time, however, the increase of the Nottingham cooling at higher temperatures ($T>T_\text {N}$) also causes the hot core to sink deeper, which smooths the temperature gradients and finally damps the feedback loop: as the emission surface evacuates more and more calories, the hot core finds its equilibrium farther from the surface. It is worth noting here that the equilibrium position along the *z*-axis is also influenced by the variation of the emitter section. Chiefly, if the cross-section of the emitter gets larger as it gets closer to the base, the Joule heating density rapidly shrinks. A stable position is therefore reached sooner, which temper the thermal jump.

The above scenario explains how exceeding the Nottingham temperature at the protrusion apex can trigger a transient Joule runaway. This runaway precisely is the cause of the temperature jump initially observed in the transition with the applied electric field towards intense thermo-field emissions (Fig. 2b). We therefore propose to name this whole mechanism the *Nottingham Inversion Instability*. The conditions for this thermal instability to occur can be summarized as follows:

1. The Nottingham effect at the emitter apex has reversed from heating to cooling.
2. The Nottingham cooling magnitude is significant and spreads over a wide emission surface, so that it considerably disturbs the temperature gradients beneath the apex.
3. The thermal conductivity is high enough so that the new gradient distribution yields a noticeable heat reflux, i.e. a heat transfer in the opposite direction to the thermostat, from the high-temperature domain (the “hot core” formed around the maximum temperature) to the emission surface.

It is also worth adding that this mechanism benefits from a steep decrease of the electrical conductivity with the temperature around the Nottingham inversion, and a geometry with a constant section along the emitter axis.

Interestingly enough, the Nottingham cooling that results from exceeding the inversion temperature has often been mentioned as thermally stabilizing the field emission from protrusions^7,25,41^, as opposed to the resistive heating which can be unstable on its own if the heat dissipation is too weak. Although this argument is sounded, it is based on 1D stationary analytical models. It should therefore be tempered with respect to the Nottingham Inversion Instability, which shows how exceeding the Nottingham temperature can be the very cause of explosive thermal failures.

An experimental work from Spindt on field emission from arrays of micrometric molybdenum cones exposes the case of a thermal failure in that sense^42^(subsection III-E therein): the retrospective micrograph highlights “a particularly violent disruption of a single cone in a 5000-cone array”, supporting no (or few) vapor release but rather liquid metal ejections around the explosion center. This would suggest that the melting temperature of molybdenum was reached *beneath* rather than *at* the emitter apex. Additionally, an early work of Dyke et al., which studied the field emission stability of a tungsten micrometric tip *versus* increasing voltage pulses, reported a reproducible current intensity jump at 1 or 2% below the actual voltage at which “electrical breakdown in the form of an explosive vacuum arc occurred”. Besides, evidences are given supporting that “temperatures greater than 2100 K are required for this effect”. The current intensity jump is significant enough to be visible on the Fowler–Nordheim plot of the data ^43^ (Fig. 3 therein, measurements E and F). Whether this jump is related to a Nottingham Inversion Instability or not cannot be certified. Still, it suggests the possibility to experimentally search signatures of the Nottingham Inversion Instability *via* Fowler–Nordheim analyses of single emitters. Such an investigation would contribute to better understand the influence of the emitter parameters on the emission stability.

Nevertheless, we are aware that research on field emitter arrays has nowadays turned much of its attention from metallic micro-tips to carbon nanostructures. Their electric and thermal properties being less conventional, their study was beyond the scope of this work. Still, considering the high sublimation temperature of graphite, we think that the Nottingham Inversion Instability can play a role in some prompt thermal failures of carbon nanostructures emitting in the thermo-field regime. Besides, carbon nanotube failures with the breaking point along their shaft has already been observed in the literature^44^, highlighting the influence of the Nottingham effect in the process.

Recent works made progress to simulate the heat transfer inside emitting single-walled or multi-walled carbon nanotubes, and even carbon nanofibers on a larger scale^45^. Yet, for various reasons, they missed the physics of the Nottingham Inversion Instability. Some explore emission regimes where they found the Nottingham effect negligible^21,46^. Others only explore steady-state solutions of the heat equation and (or) limits the voltage exploration to just a few different values^44,47–49^. We therefore suggest that such careful thermal studies should be used to further investigate the Nottingham Inversion Instability in the case of emitting carbon nanostructures. Based on our results, the model should accurately track the heat evolution, explore the transition towards intense thermo-field emission up to the pre-breakdown voltage and consider the various possible geometries of carbon nanostructures. Additionally, as the local electric field over the surface of carbon nanotubes actually is not perfectly homogeneous^50^, the thermo-field emission of carbon nanostructures should be investigated in 2D axisymmetric (or even 3D) geometries to carefully explore the consequences of the Nottingham effect at the apex. This topic will be addressed in further works.

## Conclusion

Our results unveil the theoretical possibility for a thermal instability to occur during the field emission of a micrometric refractory metal emitter when its apex temperature exceeds the Nottingham inversion temperature.

It was known that exceeding the Nottingham temperature at the emitter apex causes the maximum temperature to sink into the bulk, forming a high temperature domain—the so-called *hot core*– beneath the emission surface. Our careful study of the heat evolution in time showed how this criterion can be related to the initiation of a positive feedback loop causing a transient Joule runaway. The latter quickly brings a significant heat surplus to the emitter. It therefore precludes a whole range of thermal energy to be reached in steady states, yielding a jump in the maximum temperature variation with the applied electric field.

The runaway appears to be due to an emerging heat *reflux* (i.e. in the opposite direction to the thermostat) from the high temperature domain towards the emission surface. This is why exceeding the Nottingham temperature at the apex is a necessary condition, although not sufficient. The material thermal conductivity also plays a major role, highly affecting the amplitude of the heat reflux. Additionally, a large emission surface also benefits the heat reflux, increasing the proportion of heat being dissipated at the emission surface rather than towards the thermostat. Together, these three criteria determine the occurrence and the amplitude of the whole mechanism that we propose to define as the *Nottingham Inversion Instability*. Besides, the use of at least 2D axisymmetric models is also necessary to grasp the Nottingham heat flux variation over the emission surface. The resulting radial components of the heat reflux influence the initiation of the Nottingham Inversion Instability, as they determine the shape of the hot core. Overall, the heat surplus produced by this instability facilitates the thermal explosion of field emitters and partly hampers the benefit of refractory metal high melting temperatures, drawing closer the breakdown voltage.

On a more general note, the Nottingham Inversion Instability highlights how well-known equations can yield unexpected behaviors, impossible to resolve analytically, when coupled together on realistic geometries. It is in its way a shining example of those complex feedback mechanisms that are wiped out when physics is oversimplified.

## Material properties

For the material properties, our model uses fitting polynomials to reproduce the tabulated values from various references (see Fig. 6). The electrical conductivities are set accordingly with the values proposed by Desai et al.^51^. The thermal conductivities of molybdenum and tantalum follow the values proposed by Ho et al.^52^. Finally, the thermal conductivity of tungsten uses the slightly more recent values of Binkele^53^. Note, however, that the thermal conductivity values of tungsten above 1266 K are from Ho et al.^52^ and were multiplied by 0.84 to match the more recent data of Binkele^53^. The work function for the tungsten is taken homogeneous and equal to its polycrystalline value of 4.5 eV, in accordance with the results of Reimann^54^ and Swanson and Crouser^55^, even though the authors are aware of the significant variation depending on the crystal direction (see Swanson and Schwind^56^, table 1 therein). When the material properties are then changed in subsection 3.3, the work function is kept at 4.5 eV, in order to isolate the influence of the conductivities. Concerning the volumetric heat capacities, they are quite similar for all three materials. We used the values of White and Collocott^57^ for tungsten, Desai et al.^51^ for molybdenum and McBride^58^ for tantalum.

## Supplementary Information

Supplementary Information 1.
