# Publisher Correction: Unveiling the Nottingham Inversion Instability during the thermo-field emission from refractory metal micro-protrusions

**DOI:** 10.1038/s41598-021-98197-0

**Published:** 2021-09-13

**Authors:** Darius Mofakhami, Benjamin Seznec, Tiberiu Minea, Romaric Landfried, Philippe Testé, Philippe Dessante

**Affiliations:** 1 Laboratoire de Génie Electrique et Electronique de Paris, Université Paris-Saclay, CentraleSupélec, CNRS, 91192 Gif‑sur‑Yvette, France; 2 Laboratoire de Génie Electrique et Electronique de Paris, Sorbonne Université, CNRS, 75252 Paris, France; 3 Laboratoire de Physique des Gaz et des Plasmas, Universite Paris-Saclay, CNRS, 91405 Orsay, France

Correction to: *Scientific Reports* 10.1038/s41598-021-94443-7, published online 26 July 2021

The original version of this Article contained errors in the Introduction where,

“Let us recall the Nottingham effect comes from the energy balance between the mean energy of the emitted electrons $\left\langle {\epsilon_{{{\text{out}}}} } \right\rangle$ and that of the replacing electrons $\left\langle {\epsilon_{{{\text{in}}}} } \right\rangle ,W_{{\text{N}}} = \left\langle {\epsilon_{{{\text{in}}}} } \right\rangle - \left\langle {\epsilon_{{{\text{out}}}} } \right\rangle.$ It yields a heat flux at the metal/vacuum interface whose magnitude depends on the emitter current density *J*, $\Phi_{{\text{N}}} = - W_{{\text{N}}} \times J/e$ where *e,Nottingham* temperature and is analytically found proportional to the local electric field magnitude and inversely proportional to the square root of the emitter work function^7^: $T_{{\text{N}}} \propto F/\varphi^{1/2}.$”

now reads:

“Let us recall the Nottingham effect comes from the energy balance between the mean energy of the emitted electrons $\left\langle {\epsilon_{{{\text{out}}}} } \right\rangle$ and that of the replacing electrons $\left\langle {\epsilon_{{{\text{in}}}} } \right\rangle ,$ the so-called Nottingham energy $W_{{\text{N}}} = \left\langle {\epsilon_{{{\text{in}}}} } \right\rangle - \left\langle {\epsilon_{{{\text{out}}}} } \right\rangle.$ It yields a heat flux at the metal/vacuum interface whose magnitude depends on the emitter current density *J*, according to the formula  $\Phi_{{\text{N}}} = - W_{{\text{N}}} \times J/e$ where *e* is the elementary charge. Therefore, the sign of this heat flux can reverse. At a given field magnitude, the heat flux is positive (heating) below a certain temperature and reverses above, becoming negative (cooling). The inversion temperature is called the *Nottingham temperature* and is analytically found proportional to the local electric field magnitude and inversely proportional to the square root of the emitter work function^7^: $T_{{\text{N}}} \propto F/\varphi^{1/2}.$”

The original Article has been corrected.

