# Supplementary material for: Unveiling the Nottingham Inversion Instability during the thermo-field emission from refractory metal micro-protrusions
Source: Sci Rep. 2021 Jul 26;11:15182. doi: 10.1038/s41598-021-94443-7 (PMC8313719; doi:10.1038/s41598-021-94443-7)
Supplement: Supplementary file 1 — Supplementary Information 1. [file 41598_2021_94443_MOESM1_ESM.pdf]

# *Supplementary Material for:* "Unveiling the Nottingham Inversion Instability during the thermo-field emission from refractory metal micro-protrusions"

**Darius Mofakhami[1,2,*], Benjamin Seznec[2], Tiberiu Minea[2], Romaric Landfried[1], Philippe Teste[1], and Philippe Dessante[1]**

[1]Universite Paris-Saclay, CentraleSupelec, CNRS, Laboratoire de Genie Electrique et Electronique de Paris, 91192, Gif-sur-Yvette, France.
[2]Universite Paris-Saclay, CNRS, Laboratoire de Physique des Gaz et des Plasmas, 91405, Orsay, France.
[*]darius.mofakhami@centralesupelec.fr

The present document contains supplementary figures to support statements that were abbreviated in the original paper for the sake of conciseness.

Along with this document comes an animation: `HotCore_W_f10_Eth_delta.gif`. It shows the color map evolution in the axisymmetric plan of both the Nottingham heat flux at the emission surface and the protrusion temperature for the case of the tungsten emitter just above the threshold electric field (see Fig. 2b in the manuscript). The time spans from $128$ to $167\ \mu s$, which is right during the thermal runaway (see Fig. 3, graph d2 in the manuscript). It enables one to track in time the maximum temperature detachment and the formation of the hot core.

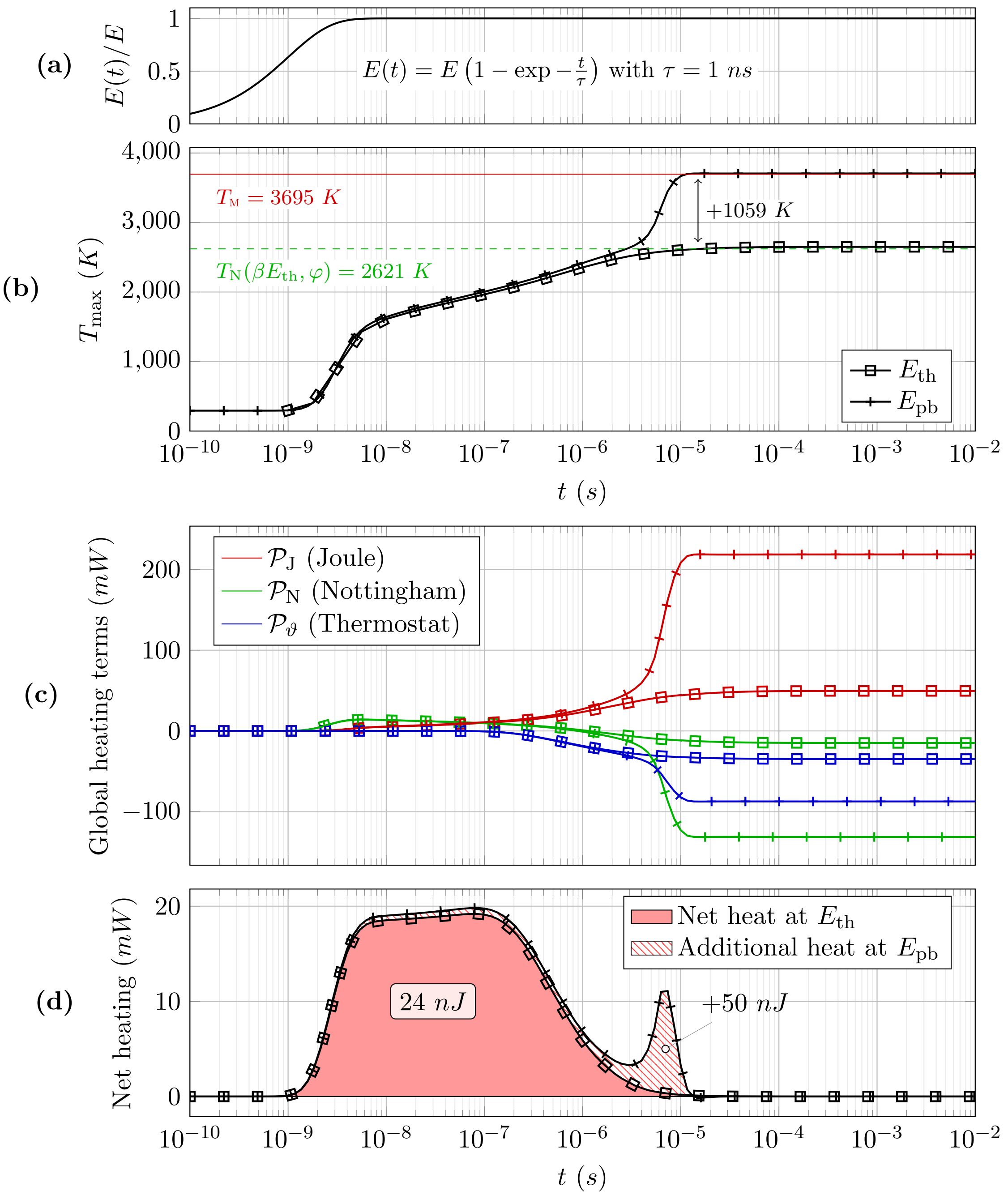


**Supplementary Figure 1.** Evolution of the self-heating process for the tungsten emitter. (a): Time ramp of the applied electric field for each simulation, normalized to 1. The time constant $\tau$ is set to one nanosecond. (b): Evolution of the maximum temperature during the self-heating process at $E_{\text{th}}$ = 201.785 $MV/m$ (square marks) and $E_{\text{pb}}$ = 202.25 $MV/m$ (plus marks). $E_{\text{th}}$ is the threshold electric field and $E_{\text{pb}}$ the pre-breakdown field. (c): Detailed evolution at $E_{\text{th}}$ and $E_{\text{pb}}$ of each global heating terms. (d): Evolution of the net heating at $E_{\text{th}}$ and $E_{\text{pb}}$. The net heating is the sum of the three global heating terms. Integrating in time the net heating yield the net heat: 24 $nJ$ at $E_{\text{th}}$ and 74 $nJ$ at $E_{\text{pb}}$, hence an additional heat of 50 $nJ$ at $E_{\text{pb}}$.

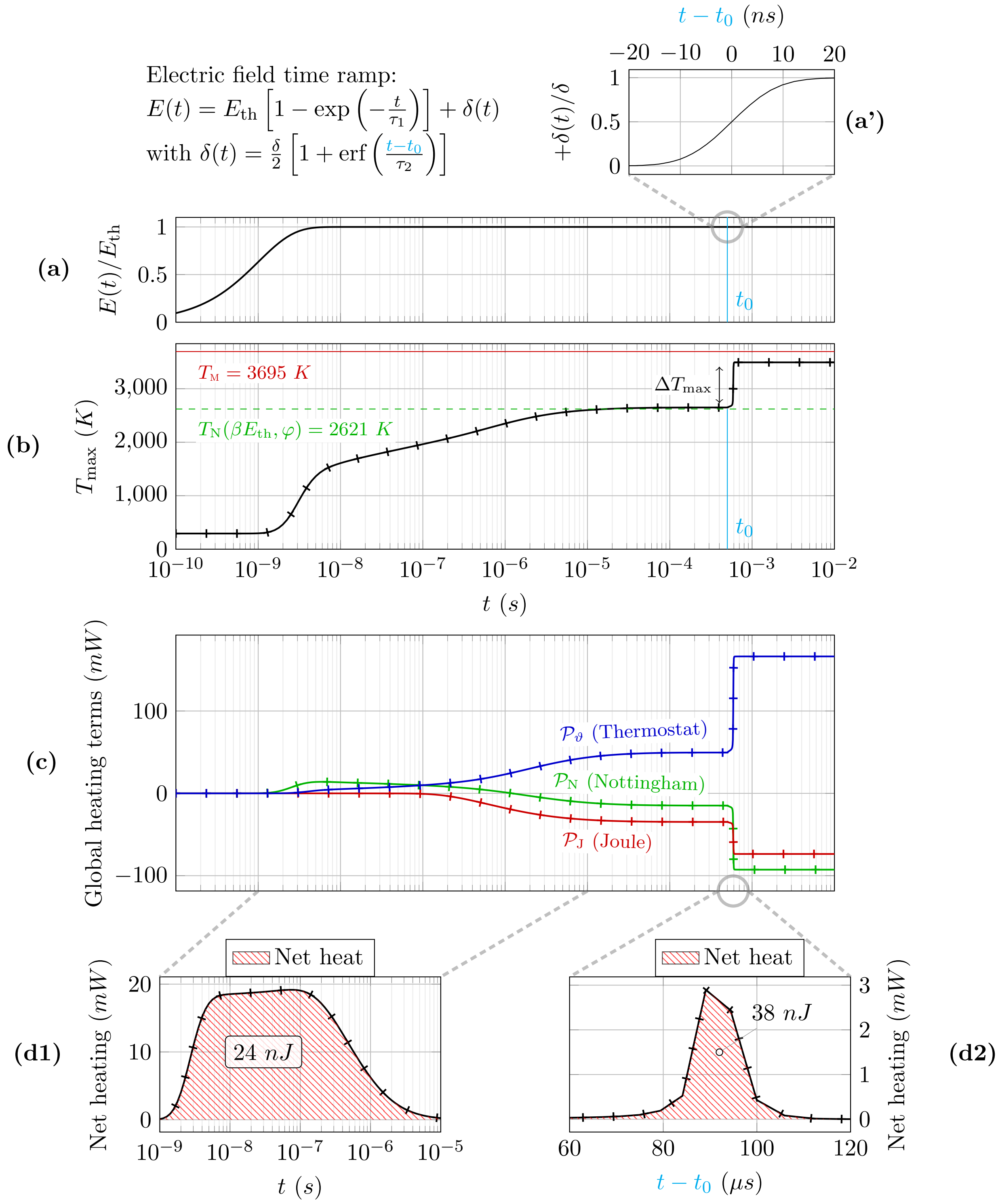


**Supplementary Figure 2.** Evolution of the self-heating process for the tungsten emitter when the electric field is ramped up by $\delta = 5\ kV/m$ at a specific time $t_0$ (highlighted in cyan), after a plateau of the maximum temperature has first been reached at $E_{\text{th}}$. **(a)**: Evolution of the applied electric field, normalized by $E_{\text{th}}$. The time constants are $\tau_1 = 1\ ns$, $\tau_2 = 10\ ns$ and $t_0 = 500\ \mu s$. **(a')** Zoom on the field increase by $\delta(t)$ around $t_0$ (linear scale). **(b)**: Evolution of the maximum temperature during the self-heating process. **(c)**: Detailed evolution of each global heating terms. **(d1)**: Net heating before the field increase by $\delta$. **(d2)**: Net heating after the field increase by $\delta$. Comparing this figure with Fig. 3 in the manuscript, we see that the same net heat values are found whether the $\delta$ increase is taken into account from the beginning, or occurs after a given time $t_0$. The thermal runaway also develops over a similar time interval (a few dozens of microseconds).

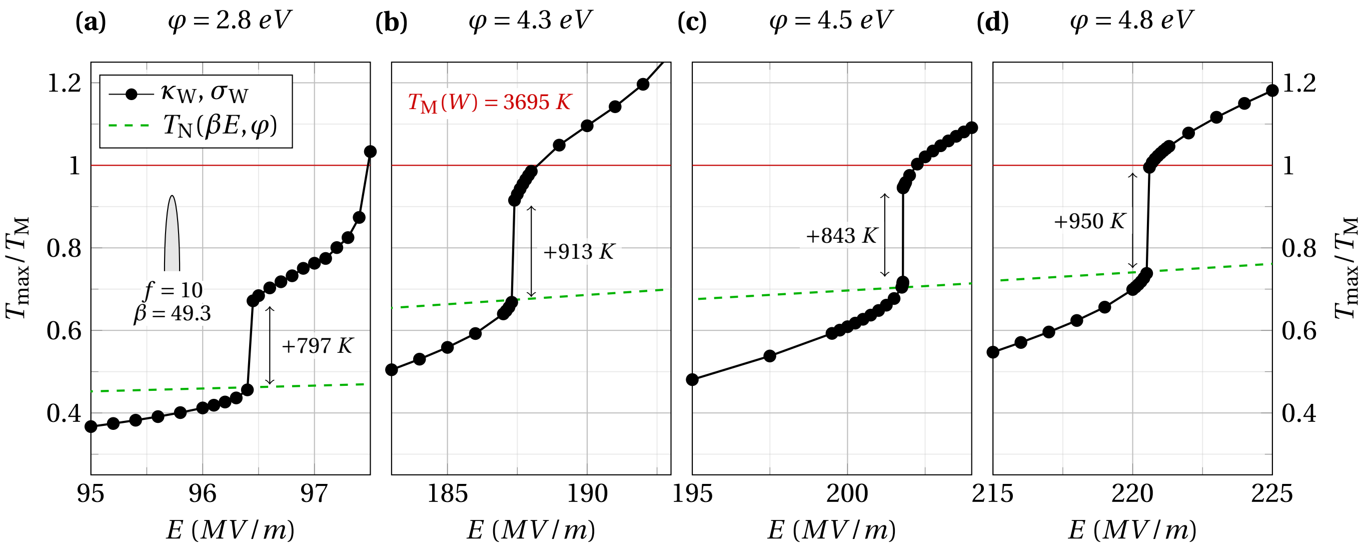


**Supplementary Figure 3.** Variation of the tungsten emitter maximum temperature with the applied electric field for four different work functions. **(a)**: $\varphi = 2.8\ eV$ corresponds to the work function value given in [1] for thoriated tungsten. Graphs **(b)**, **(c)** and **(d)** explore three different values to simulate the variation of the tungsten work function depending on the crystal directions. $\varphi = 4.3\ eV$ and $\varphi = 4.8\ eV$ respectively approximate minimum and maximum values given in [2], table 1 therein. $\varphi = 4.5\ eV$ corresponds to the polycrystalline value given in [1, 3]. The occurrence of the thermal jump is not affected by the work function value. Moreover, the maximum temperature jumps are overall similar.

## References


**1.** Reimann, A. L. The temperature variation of the work function of clean and of thoriated tungsten. *Proc. Royal Soc. London. Ser. A - Math. Phys. Sci.* **163**, 499–510, DOI: 10.1098/rspa.1937.0241 (1937).

**2.** Swanson, L. W. & Schwind, G. A. Chapter 2 A Review of the Cold-Field Electron Cathode. In *Advances in Imaging and Electron Physics*, vol. 159 of *Advances in Imaging and Electron Physics*, 63–100, DOI: 10.1016/S1076-5670(09)59002-7 (Elsevier, 2009).

**3.** Swanson, L. W. & Crouser, L. C. Total-Energy Distribution of Field-Emitted Electrons and Single-Plane Work Functions for Tungsten. *Phys. Rev.* **163**, 622–641, DOI: 10.1103/PhysRev.163.622 (1967).
